# Best clinical practise guidance for oral health care management of long-term childhood cancer survivors (CCS): an EAPD policy document

**DOI:** 10.1007/s40368-025-01037-2

**Published:** 2025-04-23

**Authors:** K. Seremidi, S. Gizani, M. Anderson, G. Dahllöf, M. Barr-Agholme, S. Parekh, G. Tsilingaridis, on behalf of European Academy of Paediatric Dentistry (EAPD)

**Affiliations:** 1 Department Paediatric Dentistry, School of Dentistry, National and Kapodistrian University of Athens, Athnes, Greece; 2 Department of Pediatric Dentistry, Public Dental Service, Eastman Institute, Stockholm, Sweden; 3 Division of Paediatric Dentistry, Department of Dental Medicine, Karolinska Institutet, 14104 Huddinge, Sweden; 4 Center of Pediatric Oral Health, Stockholm, Sweden; 5 Center for Oral Health Services and Research, Mid-Norway (TkMidt), Trondheim, Norway; 6 Department of Paediatric Dentistry, UCL Eastman Dental Institute, London, UK

**Keywords:** Childhood cancer survivors, Oral health care, Dental management, Late dental effects

## Abstract

**Purpose:**

The European Academy of Paediatric Dentistry (EAPD) has developed this best clinical practice guidance to help clinicians manage the oral health of long-term childhood cancer survivors.

**Methods:**

An expert group conducted a systematic review of the relevant literature on oral health care management of long-term childhood cancer survivors (CCS). The workshop was held during the corresponding EAPD interim seminar in Prague in May 2023. Several clinical based recommendations and statements were agreed upon, and gaps in our knowledge were identified.

**Results:**

The evidence regarding prevalence was limited to retrospective studies of moderate to good quality. Oral hygiene of CCS was worse compared to healthy individuals, showing higher values for gingival and plaque indices. Similarly, survivors had increased caries risk with higher mean dmft/DMFT and dt/DT values. The most common radiographic defects were impaired root growth in the permanent teeth and tooth agenesis. Enamel developmental defects, microdontia, and hypodontia were also commonly diagnosed. Age at start of treatment was identified as a risk factor, for the development of microdontia, tooth agenesis, and root defects in patients treated at age <3 years. The type of treatment also appeared to influence the risk, as an increased prevalence of dental caries and a higher frequency of root malformations were found in patients who had undergone concomitant radiation therapy, although evidence was limited. Treatment duration was not found to be a risk factor.

**Conclusion:**

These guidelines provide recommendations for dental management for childhood cancer survivors defined as children and adolescents up to the age of 19 years, regardless of age at initial diagnosis and treatment initiation.

## Introduction

Antineoplastic treatment aim to destroy cancer cells whilst eliminating their local and distant effects. It includes chemotherapy, radiotherapy and haemopoietic stem cell transplantation or combination of different therapeutic modalities of varying intensity, depending on factors related to both the patient and the disease (Bosse et al. 2020). Despite its therapeutic effect, antineoplastic treatment can have a negative impact on healthy tissues because both chemotherapy and radiotherapy lack specificity and cannot differentiate between neoplastic and metabolically active healthy cells. Simultaneously, given that cancer has the ability to invade adjacent tissue or to spread to distant sites with microscopic metastasis the direct effect on developing tissues is common, with an average of 75% of children presenting a late effect in any organ as a result of antineoplastic treatment (Oeffinger et al., 2006; Blaauwbroek et al. 2007).

The effect of cytotoxic agents and radiation on developing cells interfere directly or indirectly with craniofacial growth, causing profound systemic abnormalities (Cetiner et al., 2019; Effinger et al. 2014). Dental anomalies are amongst the most common long-term side effects of childhood cancer therapy. They may be considered detrimental, as teeth, unlike other hard tissue structures, such as bone, do not remodel and can have anatomical, functional and aesthetic complications (Seremidi et al. 2019), all of which can potentially impact on quality of life (Rhee et al. 2014; Yaghi-Kupelli et al., 2015). Their prevalence and severity depends on factors related to the disease and its treatment, such as age at diagnosis, the type of chemotherapeutic agents used, and site and dose of radiation if given (Gawade et al. 2014; Kang et al., 2018).

Systematic reviews have mainly focussed on the evidence regarding the long-term effects of antineoplastic treatment on dental hard tissues (Seremidi et al. 2019; Busenhart et al. 2018; Gawade et al. 2014). The clinical data regarding dental care of childhood cancer survivors (CCS) are scarce. The European Academy of Paediatric Dentistry (EAPD) proposes this best practise guidance on dental management of long-term CCS using an evidence-based approach.

## Methods

An expert group (Seremidi Kyriaki; Gizani Sotiria; Dahllӧf Gӧran; Barr-Agholme Monica; Kloukos Dimitrios; Tsilingaridis Georgios) was invited by EAPD to undertake a systematic review to evaluate existing knowledge regarding the late effects of cytotoxic protocols in oral, dental, and craniofacial development. This best practise clinical recommendation has derived from both the systematic review presented by the expert group during the 13 th EAPD interim meeting seminar in Prague in May 2023, and the subsequent discussion from the working group. The group consisted of nominated delegates from EAPD member countries, moderated by two members of the EAPD Clinical Affairs Committee (Anderson Maria; Seremidi Kyriaki).

The systematic review by Seremidi et al. 2024, investigated:

- the long-term effects of antineoplastic treatment on caries prevalence, periodontal disease, dental hard tissues,and the craniofacial complex;
- the dental and oral care of long term CCS, aged up to 20 years of age, in terms of both self/home-care measures and dental rehabilitation in practice;

The best clinical practise guidance were developed by the expert group and members of the Clinical Affairs Committee on behalf of the European Academy of Paediatric Dentistry.

## Results

### Long-term dental defects

The evidence regarding prevalence was limited to retrospective studies of moderate to good quality (based on the Newcastle Ottawa Scale (Stang 2010)). Overall results showed that:

- *Oral health:* oral hygiene of CCS was worse compared to healthy individuals, showing higher values for gingival and plaque indices. Similarly, survivors had increased caries risk with higher mean dmft/DMFT and dt/DT values.
- *Developmental dental defects:* the most common radiographic defects were impaired root growth in the permanent teeth and tooth agenesis. Enamel developmental defects, microdontia, and hypodontia were also commonly diagnosed.
- *Other defects*: salivary glands showed a slightly reduced stimulated salivary flow rate but high buffer capacity for the majority of the survivors. Also increased counts of *S. mutans* and *Lactobacilli* were reported.
- *Risk factors:* age at antineoplastic treatment was identified as a risk factor, for the development of microdontia, tooth agenesis, and root defects in patients treated at age < 3 years. The type of antineoplastic treatment also appeared to influence the risk, as an increased prevalence of dental caries and a higher frequency of root malformations were found in patients who had undergone concomitant radiation therapy, although evidence was limited. Treatment duration was not found to be a risk factor (Seremidi et al. 2024).

### Dental and oral care

Data were scarce and retrieved from case reports of poor quality (based on the Newcastle Ottawa Scale). Results showed that a typical approach involved combining restorative treatment with prosthodontic rehabilitation to enhance oral function, preserve bone structures, and improve aesthetics. Dental implants and implant-retained dentures were sometimes used, even for younger survivors, with interim prosthetic solutions adjusted for growth and emerging permanent teeth. The suitability of orthodontic treatment was uncertain, with some reports suggesting it may be contraindicated, especially in cases with severe root defects. However, in other instances, orthodontic treatment was performed to correct malocclusion. Therefore, orthodontic treatment is not contraindicated but should be decided upon in an individualised basis taking into considerations specific patients risk factors (e.g. severity of dental defects and progressive effects of the cancer).

## Challenges regarding dental management

- The psychosocial impact of antineoplastic treatment can impact a child’s overall quality of life, potentially influencing their cooperation and ability to adhere to recommendations.
- Secondary effects of antineoplastic treatment on organs (such as heart, lungs and spleen) may have implications for both oral health and the approach to dental management (e.g. immune function, the need for antibiotic prophylaxis and pulmonary function status).
- Due to risk for cancer relapse a 2-year disease-free period is recommended before proceeding with elective treatment planning.
- Early diagnosis of late effects, will allow for precise and early consultation and individualised treatment planning according to patient’s needs and risk factors.

## Conclusions

These guidelines provide recommendations for dental management for childhood cancer survivors defined as children and adolescents up to the age of 19 years, regardless of age at initial diagnosis and treatment initiation:

### Dental late effects

- Classified as systemic defects including developmental, soft tissue and other (e.g. dental hard tissues, bone) or patient-reported symptoms (e.g. xerostomia, taste disturbances).
- The type of defect is directly related to the stage of odontogenesis when the antineoplastic treatment was performed.
- The type of defect and its severity is not predictable. Age at initial diagnosis is the only definite risk factor.

### Dental and oral care

- Paediatric dentists play a vital/critical role in the oncology team and should maintain continuous communication with medical doctors to acquire comprehensive information about the child’s cancer treatment, haematological and immunological status. In addition, after the child completes cancer therapy, paediatric dentists should be promptly informed if any specific precautions are necessary for dental procedures.
- Considering these patients’ potential risk of developing oral defects, regardless of their age at presentation, pediatric dentists are responsible for ensuring a smooth transition to general dental practitioners for long-term follow-up care.
- Treatment plan should be individualised and tailored to the specific type of oral complications detected. In many instances, a multidisciplinary approach may be necessary. Treatment may involve interim rehabilitation requiring ongoing adjustments until growth is completed, particularly in young patients.

## Recommendations

**Fig. 1:**
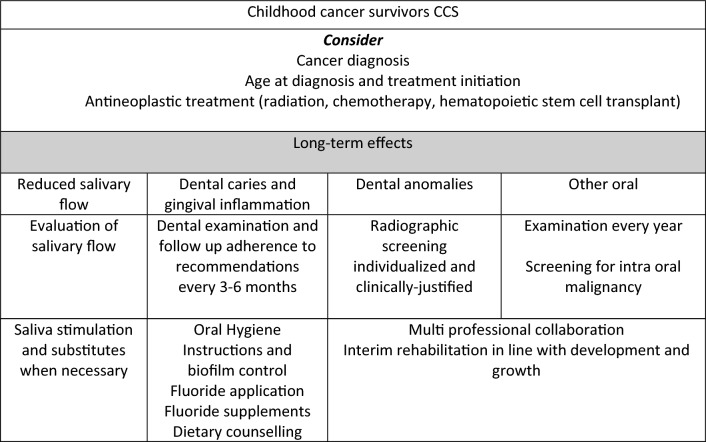
Recommendations for childhood cancer survivors in dental practise

The recommendations are summarised below and in Fig. 1.

### Dental late effects

- Development of a common checklist for recording defects is recommended to allow for long-term monitoring of the defects. The use of the Modified Dental Defect Index (DDE) is recommended to report on the overall severity of the defects on developing dentition for each patient, with higher scores indicating higher severity (Clarkson et al 1989).

### Dental and oral care

- Regular clinical reviews should be scheduled every 3–6 months to establish and maintain a healthy oral environment.
- Radiographic evaluation (panoramic radiograph) for defect detection should commence based on patient’s age at cancer treatment and clinical need, and after a latency period of more than 1 year given that tooth development is a slow process.
- Patient should undergo regular monitoring by a dentist that has satisfactory knowledge and preferably has access to a pediatric dentist, should referral for a specialist opinion be required.
- Although orthodontic treatment is not contra-indicated, it must be carefully planned to avoid the potential iatrogenic damage. The orthodontist responsible for the child’s treatment should possess experience in the management of CCS.
- Prior to any orthodontic procedures, a panoramic radiograph is mandatory for assessing the current dental status. New orthodontic treatment initiation should be delayed taking into consideration the 2-year cancer relapse period. If the child had received orthodontic treatment prior to starting cancer therapy, this history might influence the decision regarding orthodontic intervention.

## Knowledge gap

- A commonly accepted research tool should facilitate advanced research to focus on early and prompt identification of CCS at risk of the development of late complications of antineoplastic therapy.
- Studies are needed to investigate the direct association between dental late effects and disease and treatment-related factors that can be characterised as potential risk factors.
- Studies with more targeted and homogeneous samples could possibly detect the direct effect of specific treatment modalities.
- Clinical studies including pre-treatment evaluation, evaluation at the end of antineoplastic treatment and long-term monitoring of survivors to detect the actual effect of treatment on dental structures.
- Studies recording the long-term progression of these defects should be also performed to offer evidence regarding long term stability of these patients.
- Studies recording dentists’ knowledge on survivor’s dental care should be undertaken.
